# New Advances in the Development and Design of *Mycobacterium tuberculosis* Vaccines: Construction and Validation of Multi-Epitope Vaccines for Tuberculosis Prevention

**DOI:** 10.3390/biology14040417

**Published:** 2025-04-13

**Authors:** Osnat Barazani, Thomas Erdos, Raafi Chowdhury, Gursimratpreet Kaur, Vishwanath Venketaraman

**Affiliations:** 1 College of Osteopathic Medicine of the Pacific, Western University of Health Sciences, Pomona, CA 91766, USA; osnat.barazani@westernu.edu (O.B.); thomas.erdos@westernu.edu (T.E.); 2 Department of Biology, University of California, Riverside, CA 9252, USA; raafi.chowdhury@email.ucr.edu; 3 College of Pharmacy, Western University of Health Sciences, Pomona, CA 91766, USA; gursimratpreet.kaur@westernu.edu

**Keywords:** mycobacterium, *Mycobacterium tuberculosis* (Mtb), BCG vaccine, multi-epitope vaccine (MEV), PP13138R vaccine, PE_PGRS17 vaccine, Ag85A-, Ag85B-, ESAT-6-, and CFP-10-based multi-epitope vaccine

## Abstract

*Mycobacterium tuberculosis* (Mtb) vaccines are designed to prevent infection, prevent reactivation of latent infection, and/or provide adjuvant therapy to standard TB treatment for active Mtb. Emerging vaccine technologies include reverse vaccinology, DNA and RNA vaccines, subunit vaccines, and multi-epitope vaccines. Currently, many different types of vaccine candidates are in clinical trials, though, to date, BCG remains the only approved Mtb vaccine. Mtb has a complex genome with numerous antigens, but not all are equally effective in eliciting immunity, so a critical challenge is the selection of antigens and epitopes that are most likely to induce a long-term, broad-spectrum protective immune response. Multi-epitope vaccines (MEVs) represent a new event horizon in vaccine development. Bioinformatic computer modeling is being used to maximize efficacy and minimalize adverse effects. Although no multi-epitope vaccines have proceeded to in vivo clinical trials, three candidate MEVs have made it through in silico tests. Multi-epitope vaccine candidate PP13138R, containing 13 HTL epitopes, 13 CTL epitopes, and 8 B cell epitopes in addition to both TLR2 and TLR4 agonists, aims to elicit a broad immune response that could address both active and latent Mtb infection. Similarly, immunoinformatic data were used to design and validate another MEV candidate based on the biomarker PE_PGRS17 with four B cell, nine HTL, and six CTL linked epitopes, with a griselimycin sequence as the adjuvant. A third novel prophylactic and therapeutic MEV was developed that targets Ag85A, AG85B, ESAT-6, and CFP-10 proteins with 12 CTL, 25 HTL, and 21 LBL epitopes with a CpG adjuvant.

## Simple Summary

Tuberculosis (TB), caused by the bacteria *Mycobacterium tuberculosis* (Mtb), is one of the world’s deadliest infectious diseases due to its ability to evade the human immune system. Only one vaccine has ever been licensed for Mtb, but it has highly variable protection and is especially limited in adults and in high-burden areas. With drug-resistant strains of Mtb now relevant, scientists are working on developing new vaccine strategies. Multi-epitope vaccines, one of the newest vaccine technologies, utilize small sections called epitopes from the bacteria that can train the immune system to fight TB more effectively. Designed using computer programs and extensive databases to select multiple regions of the bacteria that can trigger a broad, strong, and safe immune response, these vaccines have the potential to offer customized protection for people of all ages against different forms of TB, including drug-resistant strains. Recent studies have identified several promising multi-epitope vaccine candidates that have shown strong immune activation in computer modeling and laboratory simulations. While MEVs are not in production yet or in clinical trials, they represent a cutting-edge strategy that could offer broader, more durable protection against both active and latent TB infection.

## 1. Introduction

Tuberculosis is the leading infectious disease killer in the world, with one-fourth of the global population estimated to be infected with Mtb and 1.25 million people dying from the disease in 2023 [1,2]. Of the estimated 10.8 million people who contracted tuberculosis in 2023, 55% were men, 33% were women, and 12% were children [1]. The incidence rate of Mtb has increased 4.6% since 2020 [1].

Unequally distributed around the world, Mtb is particularly prevalent in low- and middle-income countries where poverty, lack of proper hygiene, and a high incidence of HIV compromise the immune system. Globally, India, Indonesia, China, the Philippines, Pakistan, Nigeria, Bangladesh, and the Democratic Republic of the Congo have the highest disease burden, accounting for more than two-thirds of the world total [1,3].

Contagious and airborne, the disease has infected people for thousands of years; however, it was not until the late 19th century that *Mycobacterium tuberculosis* (Mtb) was identified as the causative agent [2,4]. Although Mtb was historically one of the leading causes of death in Europe and North America, where 80% of those infected died, improved living conditions, sanitation, nutrition, and public health measures produced a pronounced decline of Mtb in the developed countries of Western Europe and North and South America [2]. Consequently, investing in public health infrastructure to ensure good nutrition, clean water, sanitation, and education on hygiene is crucial to the World Health Organization’s goal of ending the Mtb epidemic by 2030 [1].

While the development of the Bacillus Calmette-Guerin (BCG) vaccine and antibiotics significantly reduced the mortality rate of Mtb, the disease remains a significant public health challenge. The pathogen has an uncanny ability to escape the human immune system; it can adapt and survive within a host for long periods and become activated years later. Mtb can also develop resistance to antibiotics with such efficiency that the disease is now divided into the subcategories of drug-sensitive TB, multi-drug-resistant TB, and extensively drug-resistant TB. As the number of Mtb cases rapidly increases [3], the limited protection the BCG vaccine provides in high-burden settings is a major concern, so the search continues for a vaccine that can provide broad, long-lasting protection in all age groups and across strains.

## 2. Mechanisms of Interaction Between *M. Tuberculosis* and the Host

One way to address Mtb’s ability to escape the immune system is to utilize multi-epitope vaccines that can help the immune system identify multiple regions of the pathogen, decreasing the chance for Mtb to evade identification. This section will discuss Mtb’s pathogenicity and involvement with the immune system as it relates to different aspects of vaccine development.

### 2.1. Innate Immune Response

The largest role in the early immune response against Mtb infection is played by macrophages and dendritic cells. This is an innate immune response that utilizes pattern recognition receptors (PRRs) to detect pathogen-associated molecular patterns (PAMPs). Relevant PRRs to Mtb vaccination include toll-like receptors (TLRs), C-type lectin receptors (CLRs), and NOD-like receptors (NLRs) expressed on various immune cells including macrophages and dendritic cells, as well as Mannose Receptor (MR), DC0SIGN, and Macrophage Receptor with Collagenous Structure (MARCO), which bind to components like lipoarabinomannan (LAM) on Mtb [5]. The activation of macrophages and dendritic cells initiates a complex immune response aimed at controlling the infection while balancing inflammatory responses [6].

TLR2 and TLR4 are added onto multi-epitope vaccines because they detect lipoproteins and glycoproteins on the Mtb surface and proceed to activate the NF-kB signaling pathway, leading to pro-inflammatory states via the production of TFF- α, IL-1β, and IL-6 [7]. Vaccines also aim to recognize unmethylated CpG motifs in Mtb DNA, which can be achieved with TLR9 (among other options) [8].

### 2.2. Adaptive Immune Response

#### 2.2.1. Activation of CD4+ and CD8+ T Cells

Even more relevant to multi-epitope vaccines is adaptive immunity. The goal of an epitope vaccine is to give the immune system the ability to recognize a particular pathogen as Mtb and then mount the appropriate response. Upon infection, Mtb antigens are processed and presented by antigen-presenting cells (APCs) via MHC class II molecules, which activate CD4+ T cells (which differentiate into various subsets, including Th1 cells) [9]. The presence of CD4+ T cells is critical for the control of Mtb, as evidenced by the increased susceptibility to tuberculosis in individuals with HIV, who have depleted CD4+ T cell counts.

CD8+ T cells are also activated during Mtb infection, although their role is less well understood compared to CD4+ T cells. CD8+ T cells recognize Mtb antigens presented by MHC class I molecules. Mtb can utilize an alternate antigen-processing pathway for MHC class I presentation, which is insensitive to conventional inhibitors like brefeldin A [10]. Activated CD8+ T cells can produce IFN-γ and exhibit cytotoxic activity, directly killing infected cells through the release of perforin and granzyme B. The activation of CD8+ T cells is also influenced by the presence of CD4+ T cells, as CD4+ T cell-derived signals are necessary for optimal IFN-γ production by CD8+ T cells.

#### 2.2.2. Formation of Granulomas

Another means by which Mtb has evaded vaccination and treatment is via granuloma formation. Granulomas are structured aggregates of immune cells, primarily composed of macrophages, including infected and uninfected macrophages, foamy macrophages, epithelioid cells, and multinucleated giant cells, surrounded by a ring of lymphocytes [11]. Macrophages engulf Mtb but are unable to completely eradicate it, leading to the formation of a granuloma and the persistence of Mtb with the potential for reactivation under certain conditions [12]. This dynamic structure is central to both the host’s defense and the pathogen’s persistence, making it a critical focus for understanding tuberculosis pathogenesis and developing therapeutic strategies. Currently, the vaccination strategy involves activating Th1 immune responses that can remodel granulomas to allow antibacterial penetration and the clearance of Mtb within the granuloma.

### 2.3. Mechanisms of Immune Evasion

#### 2.3.1. Modulation of Host Immune Signaling Pathways

Vaccine research must account for Mtb’s ability to modulate immune signaling pathways that allow it to evade host defenses and promote its survival. One key mechanism involves the toll-like receptor 2 (TLR2)-dependent activation of the extracellular signal-regulated kinase (ERK) pathway in macrophages. The ERK pathway induces anti-inflammation via IL-10 and the suppression of pro-inflammatory IL-12. This modulation inhibits Th1 polarization and IFN-γ production, thereby dampening the host’s protective immune response [13]. Additionally, Mtb secretory proteins, such as Ag85A and ESAT-6, interfere with T cell signaling. These proteins inhibit the phosphorylation of MAPKs (ERK1/2 and p38) and alter the binding of transcription factors NFAT and NF-κB, leading to defective IL-2 production and T cell unresponsiveness [14]. Mtb also targets negative regulatory pathways in macrophages. For instance, it suppresses the PI3K/AKT/mTORC1 pathway, which increases the expression of matrix metalloproteinase-1 (MMP-1) and multiple pro-inflammatory cytokines, contributing to tissue destruction and immunopathology [15]. Furthermore, the bacterium exploits the host ubiquitin system. The mycobacterial protein Rv0222 undergoes lysine-11-linked ubiquitination by the host E3 ubiquitin ligase ANAPC2, which suppresses pro-inflammatory responses by preventing the activation of TRAF6 [16].

#### 2.3.2. Suppression of Antigen Presentation

Mtb suppresses antigen expression primarily through the inhibition of major histocompatibility complex (MHC) class II-mediated antigen presentation. One of the key mechanisms involves the PE_PGRS47 protein, which inhibits autophagy in infected host phagocytes, thereby reducing the processing and presentation of antigens on MHC class II molecules [17]. This suppression leads to the decreased activation of CD4+ T cells, which are crucial for mounting an effective immune response against the pathogen. For this reason, addressing PE-PGRS proteins is a focus of epitope-based vaccines. Additionally, Mtb employs other proteins, such as PPE18, which interfere with phagolysosomal acidification, further inhibiting antigen degradation and presentation [18]. These strategies collectively enable Mtb to evade immune detection and persist within the host.

#### 2.3.3. Induction of Regulatory T Cells

While Mtb induces regulatory T cell (Treg) production through multiple mechanisms that modulate the host immune response, it is not one of the areas that epitope research has found useful to address within vaccines. The focus is instead on the safe activation of B cells, CTLs, and HTLs. 

## 3. Overview of Vaccine Development

Mtb vaccines are designed to prevent infection, prevent reactivation of latent infection, and/or provide adjuvant therapy to standard TB treatment for active Mtb. Table 1 summarizes 16 recent or ongoing vaccine trials as of August 2024 [1,19]. There were also 29 drugs in trial and at least 30 trials or studies underway for evaluating drug regimens and modes of delivery for the treatment of Mtb disease [1,19,20]. To contain the accelerating spread of multi-drug-resistant Mtb, the WHO End TB Strategy emphasizes that its goal of reaching a 90% reduction in Mtb infections and 95% reduction in Mtb deaths compared to 2015 levels can only be achieved through technological breakthroughs, including a new vaccine that is effective across all age groups, particularly adults and adolescents [1]. The WHO has defined Preferred Product Characteristics (PPCs) for new vaccines to include efficacy of at least 50%, as well as safety and efficacy in individuals with and without prior infection with *Mtb* and those with HIV infection [1,21].

## 4. Traditional Vaccine Approaches

### 4.1. Live Attenuated Whole-Cell Vaccines

#### 4.1.1. BCG

The BCG vaccine is a live attenuated form of *Mycobacterium bovis*. First used in humans in 1921, it remains the only vaccine ever approved for Mtb and is the most widely used vaccine in the world, with over 4 billion doses administered to date [2,4]. BCG has proven to be a safe vaccination without any extreme complications, and it is particularly effective for young children and infants; however, it only offers partial protection even in that group and offers no protection for adults and adolescents, though they make up the majority of the Mtb-infected population [1,2,4]. Efficacy varies from 0 to 80% across populations due possibly to exposure to nontuberculosis mycobacterium (NTM) since prior sensitization by other mycobacteria may render BCG ineffective [2]. Variable immunity levels due to population factors such as host genetics and environmental factors such as geography and HIV incidence are also proposed as explanations for BCG variability [22,23,24]. In addition, the heterogeneity of the BCG vaccine may result from variation within the substrains of the 22 strains used [2,4,25].

According to the WHO’s 2022 Global Framework for New TB vaccines, the goal is to create a replacement for the BCG Mtb vaccine that provides protection to both adults and adolescents and has an efficacy of at least 50% that will last for at least two years with a booster every five to ten years [1,3].

#### 4.1.2. VMP1002 (NCT04351685)

VPM1002, a recombinant live vaccine, is now being tested on infants [26].

#### 4.1.3. MTBVAC (NCT04975178)

An attenuated live Mtb strain genetically engineered to incorporate two independent unmarked stable deletion mutations in the virulence genes phoP and fadD26, MTBVAC includes RD1, absent in BCG [2,25].

#### 4.1.4. Pre-Travel BCG (NCT04453293)

The TIPI trial is designed to determine whether a single dose of the live attenuated BCG vaccine prior to travel to a high-burden country can lower the risk of infection in adults. The BCG being used is the Tokyo 172 strain [26].

### 4.2. Inactivated Whole-Cell Vaccines

Inactivated vaccines made from a form of the pathogen that has been killed with heat, chemicals, or radiation triggers an immune response when the immune system recognizes them as foreign invaders. Because they cannot cause disease, such vaccines would be safer for immunocompromised people [25].

#### 4.2.1. DAR-901 (NCT02712424)

The DAR-901 vaccine, made of inactivated *M. obuense*, is a scalable manufacturing process for the SRL 172 vaccine. A Phase 2 trial revealed that the vaccine was safe and well tolerated, but that it was unable to prevent infection in 13–15-year-old adolescents who had been previously vaccinated with BCG as infants [25,27]. However, those who eventually tested positive for Mtb demonstrated enhanced immune responses to ESAT-6.

#### 4.2.2. IMMUVAC (CTRI/2019/01/017026)

This vaccine was first developed for use against leprosy, but the inactivated agent, Mycobaterium, indicus pranii, may also be effective against Mtb due the similarities between their antigens. Although the cure rate between the vaccine and placebo groups was not significant, all of the individuals who were already resistant to two or three drugs and received the vaccine were cured (based on a sputum culture), compared to 76% of those who received the placebo [28].

#### 4.2.3. RUTI

RUTI, made from liposomal fragments of Mtb, and V7, inactivated M. vaccae delivered orally, are being tested as therapeutic adjuncts to traditional antibiotic treatment [25].

## 5. Emerging Vaccine Technologies

### 5.1. Reverse Vaccinology

Traditionally, vaccines were developed by isolating pathogens, growing them in culture, inactivating or attenuating them, and then testing these preparations for their ability to induce protective immunity; however, the method can be time-consuming, expensive, and dangerous. Instead of starting with the whole pathogen, reverse vaccinology sequences the pathogen’s genome and then uses bioinformatics and immunogenicity tools to predict which proteins might be good candidates based on characteristics such as antigenicity, hydrophilicity, flexibility, accessibility, turns, surface exposure, polarity, and conservation across strains to achieve broad protection. The lack of similarity to human proteins is assessed to avoid autoimmune reactions. Although the process requires sophisticated software and expertise to predict and select antigens accurately, it has the advantage of allowing for the consideration of all potential antigens, including those as yet unknown [4].

Multi-epitope vaccine construction uses reverse vaccinology by starting with the pathogen’s genome and using tools that are in silico to predict and manage different epitopes from antigenic proteins that account for MHC compatibility that is varied and cross-protected. With this approach, vaccine developments become faster, more effective, less costly, and more safe [4,29,30,31].

### 5.2. DNA and RNA Vaccines

DNA vaccines introduce plasmid DNA encoding for TB antigens, which cells then use to produce proteins, stimulating immunity, while mRNA vaccines use messenger RNA to instruct cells to produce a TB antigen protein that triggers an immune response. DNA vaccines have bacterial plasmids that encode for Mtb antigens. With these plasmids, the DNA molecules are embodied into host cells and proteins are expressed and presented on the cell surface, which stimulates humor and cellular immunity [4]. Additionally, RNA vaccines introduce mRNA into certain host cells, which uses the host cells’ transcript to synthesize the encoded tuberculosis antigen. This newly synthesized protein is then dispensed to the immune system, initiating a response [4].

Certain RNA-based vaccine candidates like BNT164a1 and BNT164b1 (NCT05537038) are currently undergoing Phase 1 clinical trials concurrently to evaluate safety and dosing regimens within a three-dose schedule [4]. They are being tested independently but in parallel in the same study to evaluate dosage levels in a three-dose schedule.

### 5.3. Subunit Vaccines

Subunit vaccines incorporate a few key whole-protein antigens that can induce an immune response without causing disease as well as an adjuvant.

#### 5.3.1. M72/AS01E (NCT06062238)

The M72/AS01E subunit vaccine candidate, which has been in development since the early 2000s, is composed of the immunogenic fusion protein M72 derived from Mtb antigens MTB32A and MTB39A and a proprietary adjuvant known as AS01E. The Phase 2b proof-of-concept trial resulted in 50% protection against progression to active pulmonary tuberculosis for three years in infected HIV-negative adults with latent Mtb, which the Gates Medical Research Institute (MRI) reports as unprecedented in decades of research [1,32]. Those in the treatment group were given two doses one month apart. At the end of the three-year study period, of the 3573 participants, 13 people in the treatment group contracted active Mtb compared to 26 in the placebo group.

The vaccine is now in a Phase 3 clinical trial that includes 26,000 people to further assess the efficacy of M72/AS01E in adults and older adolescents with latent Mtb, as well as to assess the safety and immunogenicity of the vaccine in adults and adolescents who have not been previously exposed to Mtb and those with HIV [1,26,32].

#### 5.3.2. ID93 + GLA-SE

ID93 has four Mtb antigens combined with a TLR-4 agonist adjuvant to improve the magnitude and quality of immune responses. The vaccine showed an acceptable safety profile with participants exhibiting higher humoral and Th1 cellular immune responses compared to those who received a placebo [33].

#### 5.3.3. GamTBVac (NCT04975737)

This subunit recombinant vaccine in a Phase 3 clinical trial contains two fusion proteins combining Ag85A, ESAT-6, and CFP-10 antigens with an adjuvant that contains a DEAE-dextran core and CpG oligodeoxynucleotides that serve as TLR-9 agonists. It aims to prevent Mtb in healthy adults [34].

#### 5.3.4. H56: IC31 (NCT02375698)

This subunit vaccine candidate contains an H56 antigen fusion protein consisting of three mycobacterial antigens (the early secreted antigens Ag85B and ESAT-6 and the latency antigen Rv2660c), and is intended to be used as a two-dose regimen to prevent the recurrence of drug-susceptible, uncomplicated pulmonary Mtb in individuals recently treated for Mtb infection for 4–5 months. In a Phase 1 clinical trial of 22 adults, it showed acceptable safety and produced a CD4+ T cell response predominantly for ESAT-6, but also for Ag85B. Although there was some evidence of CD8+ T cell responses for Ag85B and ESAT-6, they were smaller than the CD4+ responses. The response persisted for 6 months after the second dose [35].

### 5.4. Multi-Epitope Vaccines

Although no multi-epitope vaccines have proceeded to clinical trials on animals or humans, they are on the event horizon. Multi-epitope vaccines (MEVs) attempt to achieve the broadest response across ages and regional differences in pathogen strains by incorporating many times more antigens than are used in subunit vaccines by splicing together only the smaller epitope peptide chain sequences of the antigens. An effective multi-epitope vaccine would elicit a strong immune response without inducing the onset of autoimmune disease. In addition to provoking a broad immune response and long-lasting immunity, they could potentially be customized for specific pathogens. However, the development of these vaccines is fraught with scientific, technical, logistical, and economic challenges.

Multi-epitope vaccine construction utilizes reverse vaccinology, by starting with the pathogen’s genome and using an in silico process to identify and predict multiple epitopes from antigenic proteins that can account for varied MHC profile compatibility, cross-protection, and precision medicine [29,36]. Multiple computational software and database bioinformatic and immunoinformatic tools are being used to more quickly and efficiently predict, design, and optimize vaccine candidates without the need for physical experiments until later stages.

Selected epitopes are then assessed for their immunogenicity through the use of bioinformatic and immunoinformatic tools. The ability of the epitopes to elicit a strong immune response must then be validated experimentally, but since the use of reverse vaccinology attempts to improve upon the conventional approach to vaccine development by bypassing the cultivation stage of the pathogen to identify these antigens, this approach makes vaccine development faster, more effective, safer, and less costly [4,29,30,31].

An ideal multi-epitope vaccine candidate would possess both the ability to trigger a humoral and cell-mediated immune response, termed immunogenicity, as well as the ability to interact with the products of the immune response often in the form of antibodies or cell surface receptors, termed antigenicity [4,29,30,31,37]. The candidates should be able to do this while also being non-toxic, non-sensitive, non-allergenic, and without leading to an autoimmune disorder. Epitope candidates should also possess a strong MHC binding affinity to both MHC I and MHC II molecules. Conservation, stability, and solubility are also factors that may be taken into consideration, as well as the epitopes’ ability to protect against different human leukocyte antigen (HLA) alleles, known as population coverage [30,31]. These epitopes should be able to induce B cells, cytotoxic T cells, and helper T cells in addition to CD4 and CD8 T lymphocytes, to induce a more protective immune response [31,38,39], so the potential epitopes must first be tested for these properties, after which there is B cell, cytotoxic T cell (CTL), and helper T cell (HTL) analysis for those epitopes that have met the criteria. Adjuvants are added to improve the vaccine’s immunogenicity, which are also predicted using algorithmic tools; linker sequences bind everything together [30,31,40]. After the MEV is constructed, it is docked with a toll-like receptor because it is the interaction of an antigen with a receptor that elicits an immune response [30,31,40].

The following steps summarize the process along with some of the available computer modeling tools (see Table 2 for a list of bioinformatic and immunoinformatic tools and their functions):

- Analyze the pathogen’s genome or proteome;
- Predict T cell and B cell epitopes that are likely to be recognized by the immune system as well as the binding affinity of peptides to various MHC alleles;
- Select epitopes conserved across different strains of the pathogen to ensure broad coverage without overstimulating the immune system;
- Design a comprehensive vaccine by engineering selected epitopes into a single construct using spacers or linkers to maintain their structural integrity and immunogenicity;
- Create a 3D molecular model to ensure correct folding and presentation;
- Simulate immune responses such as antibody production, T cell activation, and cytokine production;
- Refine and optimize the vaccine based on simulation results;
- Validate the design against known immunological principles and existing data on similar vaccines;
- Perform in silico testing;
- Physically produce the optimized vaccine;
- Conduct in vitro tests;
- Perform clinical trial with animals and humans.

**Table 2:** Bioinformatic and immunoinformatic tools employed for multi-epitope vaccine construction.

Database/Server	Prediction/Function	Purpose in TB Research
IEDB (Immune Epitope Database)	Predicts and catalogs B cell and T cell epitopes	Central repository for experimentally validated and predicted TB epitopes; aids in epitope identification
ABCpred	Predicts linear B cell epitopes using artificial neural networks	Identifies B cell epitopes in *Mycobacterium tuberculosis* (Mtb) proteins for vaccine design
BepiPred	Predicts linear B cell epitopes	Used to identify potential B cell epitopes in Mtb proteins for vaccine design
ElliPro	Predicts conformational B cell epitopes	Identifies conformational B cell epitopes in Mtb proteins, essential for developing effective vaccines
MycoBrowser Database	Annotated genome database for *Mycobacterium tuberculosis*	Provides comprehensive information on Mtb proteins, including structure, function, and interactions
NetCTL 1.2	Predicts cytotoxic T lymphocyte (CTL) epitopes	Identifies CTL epitopes from Mtb proteins by integrating MHC class I binding, proteasomal cleavage, and TAP transport efficiency
IEDB MHC II Server	Predicts MHC class II binding peptides	Identifies helper T cell (HTL) epitopes from Mtb proteins for inclusion in multi-epitope vaccines
IEDB MHC I Server	Predicts MHC class I binding peptides	Identifies CTL epitopes from Mtb proteins for inclusion in vaccine design
IEDB Immunogenicity Server	Predicts the immunogenicity of MHC class I epitopes	Evaluates the immunogenic potential of Mtb-derived CTL epitopes, aiding in the selection of potent vaccine candidates
VaxiJen v2.0 Server	Predicts antigenicity	Assesses the antigenicity of Mtb proteins and peptides to prioritize candidates for vaccine development
ANTIGENpro Server	Predicts protein antigenicity	Estimates the antigenicity of Mtb proteins, guiding the selection of immunogenic targets for vaccines
AllerTOP v2.0	Predicts protein antigenicity	Ensures Mtb epitopes and vaccine candidates are non-allergic
Allergen FP v1.0 Server	Predicts allergenicity based on physicochemical properties	Provides additional assessment of allergenicity for Mtb-derived vaccine candidates
ToxinPred Server	Predicts toxicity of peptides	Ensures selected Mtb epitopes are non-toxic for use in vaccines
ExPASy ProtParam Server	Computes various physical and chemical parameters for proteins	Analyzes the stability, solubility, and other properties of Mtb proteins, important for vaccine formulation
IFN-gamma Epitope Server	Predicts IFN-gamma-inducing epitopes	Identifies Mtb epitopes that can stimulate IFN-gamma production, enhancing vaccine efficacy by promoting a Th1 response
NovoPro Server	Protein expression and purification service	Assists in the production and purification of Mtb proteins for experimental validation and vaccine development
Protein-Sol Server	Predicts protein solubility	Evaluates the solubility of Mtb proteins, important for designing stable vaccine formulations
SOLpro	Predicts the solubility of proteins in water	Similar to Protein-Sol, used to ensure that Mtb proteins selected for vaccines are soluble and stable
RaptorX Property	Predicts protein secondary structure, disorder, and solvent accessibility	Provides structural information on Mtb proteins, aiding in the design of epitope-based vaccines
PRISPRED	Predicts protein secondary structure	Similar to RaptorX, used to predict the structure of Mtb proteins for better epitope selection
I-TASSER Server	Protein structure and function prediction	Used to predict the 3D structure of Mtb proteins, aiding in the identification of conformational epitopes
GalaxyRefine Web Server	Refinement of protein structures	Refines the predicted structures of Mtb proteins to improve the accuracy of epitope mapping
ProSA-Web Server	Protein structure and validation	Validates the predicted 3D structures of Mtb proteins, ensuring accurate modeling for vaccine development
ERRAT Web-Server	Structure validation tool	Analyzes the quality of Mtb protein structures, used in the validation of vaccine candidates
RAMPAGE Web-Server	Ramachandran plot analysis for protein structure validation	Assesses the quality of Mtb protein models by evaluating their backbone dihedral angles, important for epitope modeling
Ramachandran Plot Server	Protein structure validation	Similar to RAMPAGE, used to validate the 3D structures of Mtb proteins in vaccine research
Protein Data Bank (PDB)	Repository of 3D structural data of large biological molecules	Provides structural data of Mtb proteins, essential for understanding epitope presentation
HADDOCK Server	Protein–protein docking	Simulates interactions between Mtb proteins and human immune molecules, aiding in epitope identification
PatchDock Server	Protein–protein docking	Used for molecular docking studies to analyze interactions between Mtb antigens and immune receptors
FireDock Server	Refinement of docking results	Refines docking results from PatchDock to better understand Mtb epitope interactions with immune cells
HawDock Server	Protein–protein docking refinement	Similar to FireDock, used for redefining interactions between Mtb proteins and human immune molecules
iMODS Web-Server	Protein flexibility and motion analysis	Analyzes the dynamic behavior of Mtb proteins, important for understanding epitope exposure and recognition
Java Codon Adaptation Tool Server (JCAT)	Optimizes codon usage for expression in different hosts	Optimizes the expression of Mtb proteins in different host systems, aiding in the production of vaccine components
SnapGene Software	Molecular biology software for DNA construct design	Used for designing and visualizing plasmid constructs, important in the development of recombinant Mtb vaccines
Allele Frequency Database	Provides frequency data for different alleles	Helps in understanding the distribution of Mtb epitopes in diverse populations
Protein BLAST	Finds regions of local similarity between sequences	Used to compare Mtb protein sequences with other sequences, aiding in identifying conserved and unique epitopes
NCBI Molecule Modeling Database	Provides models of protein structures	Offers structural models of Mtb proteins for use in vaccine design
Optimizer Server	Optimizes gene sequences for better expression in a host	Used to optimize Mtb gene sequences for enhanced expression in vaccine production systems
DEG (Database for Essential Genes)	Contains essential genes of organisms	Helps identify essential Mtb genes that are critical for survival and thus good targets for vaccines
CD-HIT	Clusters sequences to reduce redundancy	Used to cluster Mtb protein sequences, aiding in the identification of unique vaccine targets
BlastP	Protein–protein BLAST	Compares Mtb protein sequences to other protein sequences to identify potential vaccine targets
PROSITE	Predicts protein domains and motifs	Used to identify functional domains and motifs in Mtb proteins, crucial for vaccine design
TMHMM	Predicts transmembrane helices in proteins	Identifies transmembrane regions in Mtb proteins, useful for selecting surface-exposed epitopes
SignalP	Predicts signal peptides	Identifies secreted proteins in Mtb, which are often targeted by the immune system and are important for vaccine design
VFDB (Virulence Factor Database)	Catalogs known virulence factors in bacterial pathogens	Identifies virulence factors in Mtb, which are potential targets for vaccine development
SPAAN	Predicts adhesins and adhesin-like proteins	Identifies adhesins in Mtb, which are important for bacterial attachment and invasion, making them good vaccine targets
Pfam	Protein family database	Provides function and structural annotations for Mtb proteins, aiding in the selection of vaccine targets
GROMACS	Molecular dynamics simulation software	Stimulates the behavior of Mtb proteins and protein–protein interactions at the molecular level, aiding in epitope stability
MM-PBSA/MM-GBSA	Free energy calculations for protein–ligand binding	Calculates the binding of free energies of Mtb epitopes with MHC molecules or antibodies, which is useful for vaccine design
JCAT	Codon optimization tool	Optimizes codon usage for Mtb proteins to enhance their expression in different host organisms, critical for vaccine production

Three multi-epitope vaccine candidates have emerged.

#### 5.4.1. PP13138R Multi-Epitope Vaccine [31]

Multi-epitope vaccine PP13138R, containing 13 HTL epitopes, 13 CTL epitopes, and 8 B cell epitopes in addition to both TLR2 and TLR4 agonists, aims to elicit a broad immune response that could address both active and latent Mtb infection by stimulating various components of the immune system. It is a modification of the HP13138PB MEV that only contained a TLR2 agonist. While it is still in the preclinical stages of development, in silico testing revealed excellent antigenicity, immunogenicity, and solubility without signs of toxicity or allergenicity. This suggests that the vaccine could effectively stimulate an immune response without causing adverse reactions typically associated with allergenic or toxic substances. Additionally, PP13138R interacts strongly with TLR2 and TLR4, which is crucial for stimulating both the innate and adaptive immune responses that produce antigen-specific antibodies and cytokines. The vaccine was predicted to induce high levels of the critical cytokines IFN-γ, TNF-α, IL-6, and IL-10, a cytokine profile indicative of a robust immune response, which would enhance both cellular and humoral immunity. Analysis of the secondary structure of the vaccine and molecular docking studies showed favorable interactions, suggesting good stability and binding affinity with immune receptors. Molecular dynamics simulations further supported the stability of the vaccine construct when interacting with TLRs, which is vital for its functional efficacy. Subsequent in vitro validation showed an increase in IFN-γ+ T lymphocytes and cytokine production in different groups, indicating its potential to work across various infection stages. Real-world efficacy, safety in diverse populations, and practical aspects of production and delivery would require extensive clinical trials beyond the scope of in silico predictions.

#### 5.4.2. PE_PGRS17 Biomarker-Based Multi-Epitope Vaccine [29]

The PE_PGRS gene family in the Mtb genome is believed to play an important role in the pathogenesis of Mtb’s ability to evade the host immune system. From it, CTL, HTL, and B cell epitopes were predicted and sequenced using immunoinformatic techniques. A GPGPG linker was used to link four B cell and nine HTL epitopes, while AAY linkers were used for six CTL epitopes. The griselimycin sequence was chosen as the adjuvant to increase the immunogenicity of the vaccine and linked to the vaccine using EAAAK. Antigenicity, allergenicity, toxicity, solubility, stability, hydrophilic potential, and aliphatic nature were tested in silico. All measurements were within prescribed ranges. Secondary and tertiary structures were determined. The final molecule was 361 amino acids long, with a molecular weight of 34,341 Daltons, which was slightly under the ideal weight of 40–50,000 Daltons that is believed to be ideal for uptake by the lymphatic system.

#### 5.4.3. Ag85A-, Ag85B-, ESAT-6-, and CFP-10-Based Multi-Epitope Vaccine [41]

In Indonesia, which has the world’s second highest Mtb burden, a novel multiepitope vaccine based on Ag85A, Ag85B, ESAT-6, and CFP-10 proteins has been assembled that incorporates 12 CTL, 25 HTL, and 21 LBL epitopes to trigger robust humoral and cellular immune responses. Its comprehensive cornucopia of CTL, HTL, and LBL epitopes offers both therapeutic and preventative benefits. The CpG adjuvants provoke a response strong enough to provide long-lasting benefits by activating B cells and DCs. The selected GPGPG, KK, and AAY linkers separate inter-epitopes, enhance solubility, and prevent cross-linking to enhance immunogenicity and epitope presentation [31].

## 6. Summary of Vaccine Platforms: Advantages and Disadvantages

1. Live attenuated whole-cell pulmonary TB vaccines such as BCG elicit broad T cell immunity with 50–80% efficacy in children; however, their efficacy in adults is highly variable. In endemic areas, efficacy can be 0%, possibly due to immune desensitization from prior mycobacterial exposure. Disseminated infection in immunocompromised individuals restricts its use in high HIV settings.
2. Inactivated whole-cell pulmonary Mtb vaccines have a high safety profile since their non-replicating nature eliminates infection risk; however, the trade-off is efficacy. They elicit primarily humoral immunity, which is insufficient against pulmonary Mtb, which require strong cell-mediated CD4+ and CD8+ T cell responses for alveolar macrophage activation.
3. DNA and RNA pulmonary Mtb vaccines offer strong immunogenicity since they provoke both humoral and cellular-mediated immunity without the risk of live pathogens. Although they can be developed rapidly and are highly adaptable to target different strains, their clinical efficacy remains unproven. RNA vaccines require ultra-low storage temperatures, while DNA vaccines need optimized delivery systems.
4. Subunit pulmonary Mtb vaccines utilize specific antigens to ensure safety and avoid replication risks. Their narrow focus may be effective in some populations, but it may fail to address Mtb’s genetic diversity or latency and, thus, produce weaker cellular immunity. In addition, they require adjuvants’ support, which can be problematic in and of itself.
5. Multi-epitope pulmonary Mtb vaccines incorporate a wider variety of epitopes than the subunit vaccines to optimize coverage as a way to address Mtb’s variability. Development complexity arises from variable immunogenicity across epitopes, however, and the vaccines remain theoretical and preclinical with none in production or in trial.

## 7. Challenges and Future Directions

### 7.1. Scientific and Technological Challenges

#### 7.1.1. Antigen Selection and Epitope Mapping

A critical challenge in developing multi-epitope vaccines is the selection of antigens and epitopes that are most likely to induce a protective immune response. Mtb has a complex genome with numerous antigens, but not all are equally effective in eliciting immunity. Identifying epitopes that are broadly recognized across diverse human populations and HLA haplotypes is difficult, and there is a risk of selecting epitopes that might not confer adequate protection or, worse, induce immune evasion by the pathogen.

#### 7.1.2. Immune Response and Vaccine Design

Another challenge lies in designing a vaccine that induces a balanced and effective immune response. Mtb is a highly adaptable pathogen that can evade the immune system. Multi-epitope vaccines must induce a strong and lasting T-cell response, particularly involving CD4+ and CD8+ T cells, which are crucial for controlling intracellular infections like TB. However, achieving this requires careful vaccine design, including the choice of delivery systems, adjuvants, and formulation strategies to ensure that the epitopes are presented in an immunogenic context.

#### 7.1.3. Preclinical and Clinical Evaluation

The translation of promising in silico and preclinical findings into human trials has proven challenging. Animal models, such as mice and non-human primates, are used extensively in TB research, but they do not always accurately predict human immune responses. The complexity of TB pathogenesis, coupled with the variability in immune responses between individuals and populations, complicates the design and interpretation of clinical trials. Furthermore, establishing correlates of protection—immune markers that predict vaccine efficacy—remains an unmet need in TB vaccine research.

#### 7.1.4. Logistical Challenges

Meeting the extensive safety, immunogenicity, and efficacy data required for regulatory approval is the first logistical challenge. Scaling up the complex manufacturing processes involved in the production of multi-epitope vaccines to produce sufficient quantities for global distribution is the next challenge. Ensuring the effectiveness and stability of multi-epitope vaccines during manufacturing, storage, and transport requires adherence to a stringent cold chain, which is a significant challenge in targeted low- and middle-income countries that often do not have sufficient cold chain infrastructure for distribution. The fact that these are often the countries with the highest Mtb disease burden compounds the problem.

#### 7.1.5. Economic Challenges

The development and production of multi-epitope vaccines is a resource-intensive, highly technical process requiring skilled labor and large-scale manufacturing facilities. Peptide synthesis, recombinant protein production, and advanced adjuvant system development are expensive, particularly for deployment in the resource-limited settings that TB primarily affects. As a result, multi-epitope vaccine development relies on fluctuating funding from international organizations, philanthropic foundations, and public–private partnerships, while having to compete with other Mtb vaccine technologies for funding, as well as other diseases that may present higher burdens. Affordability and accessibility are important considerations as vaccine pricing must allow for the recovery of development costs and enough profit to incentivize development, while ensuring that the vaccine is affordable for LMICs.

## 8. Conclusions

A next-generation TB vaccine for achieving the WHO’s End TB Strategy goals has become scientifically feasible through advances in immunoinformatics and reverse vaccinology. Enabled by the computer-guided selection of epitopes, delivery mechanisms, and linkages, MEVs are designed to optimize both innate and adaptive immunity through the activation of B cells, helper T cells, and cytotoxic T cells, to create broad immunogenicity and cross-strain protection. Three MEV candidates demonstrate strong theoretical antigenicity, solubility, and favorable cytokine profiles; however, significant challenges remain in transitioning these candidates to clinical development. Future efforts must focus on validating these constructs through rigorous preclinical and clinical trials while addressing logistical barriers to ensure global accessibility. 

## Figures and Tables

**Table 1:** New vaccine trials [1,19,20].

Vaccine Name	Trial Phase	Topic	Location	Cohort Size	End Date
DAR-901 Booster	2	Randomized placebo controlled double-blind study of the prevention of infection using Mycobacterial inactivated whole-cell or extract vaccine among adolescents who have previously received BCG	Tanzania	625	November 2019
ID93+GLA-SE or QTP101	2a	Randomized double-blind placebo-controlled study to evaluate the safety, immunogenicity, and efficacy of a protein + adjuvant vaccine in BCG-vaccinated healthy healthcare workers	Republic of Korea	107	May 2020
AdHu5Ag85A	1	Safety and immunogenicity of a viral vector adenovirus-based TB vaccine administered by aerosol	Canada	36	August 2021
ChAdOx185A-MVA85A	2a	Dose-escalation and age-de-escalation study in healthy adults and adolescents to provide safety data for viral vector live vaccine	Uganda and United Kingdom	71	December 2021
MIP/Immunvac	3	Efficacy and safety evaluation of Mycobacterial inactivated whole-cell or extract vaccine in preventing TB disease among healthy household contacts of sputum smear-positive Mtb patients	India	12,000	December 2021
VPM1002	3	Efficacy and safety evaluation of recombinant live attenuated vaccine in preventing TB disease among healthy household contacts of sputum smear-positive Mtb patients	India and Bangladesh	12,000	December 2021
TB/FLU-05E	1	Randomized double-blind placebo-controlled trial of intranasal viral vector vaccine for the prevention of Mtb infection in BCG-vaccinated adult volunteers	Russian Federation	51	September 2023
AEC/BC02	2a	Safety tolerability and immunogenicity of recombinant protein + adjuvant Mtb vaccine	China	200	November 2024
BCG-Travel vaccine	3	Prevent infection using live attenuated BCG booster vaccine in those traveling to high-burden TB countries—TIPI	United States	2000	April 2025
VPM1002—infants	3	Evaluation of the efficacy and safety of live attenuated VPM1002 in the prevention of Mtb infection among infants in comparison to BCG	Gabon	6940	October 2025
GamTBvac	3	Safety and efficacy study of the subunit recombinant vaccine with adjuvant to prevent Mtb infection	Russian Federation	7180	October 2025
RUTI	2b	Investigate the therapeutic and prevention capability of inactivated Mycobacterial whole-cell or extract vaccine	India and Argentina	140	November 2025
BNT 164a1 BNT 164b1	1	Safety and immunogenicity of two investigational RNA-based Mtb vaccines	Germany	96	February 2026
H107e/CAF 10b	1a	Dose-finding and open-label trial followed by a Phase 1b, double-blind, randomized, and placebo-controlled trial to evaluate the safety, reactogenicity, and immunogenicity of a protein + adjuvant subunit vaccine in adults	South Africa	140	May 2026
MTBVAC	3	Efficacy, safety, and immunogenicity of a live attenuated vaccine administered in healthy HIV-unexposed and HIV-exposed uninfected newborns in TB-endemic regions of Sub-Saharan Africa	South Africa	6960	August 2029
M72/AS01E	3	Randomized, double-blind, placebo-controlled, and multicenter clinical trial to assess the prophylactic efficacy, safety, and immunogenicity of a protein + adjuvant subunit vaccine	South Africa	26,000	August 2029

## Data Availability

No new data were created or analyzed in this study.
